# Pathogen Identification, Antagonistic Microbe Screening, and Biocontrol Strategies for *Aconitum carmichaelii* Root Rot

**DOI:** 10.3390/microorganisms13092202

**Published:** 2025-09-19

**Authors:** Xingxun Dai, Yuqin He, Yu Su, Huishu Mo, Weichun Li, Wanting Li, Shuhui Zi, Lufeng Liu, Yining Di

**Affiliations:** 1 College of Resources and Environment, Yunnan Agricultural University, Kunming 650201, China; 2 College of Agronomy and Biotechnology, Yunnan Agricultural University, Kunming 650201, China; 3 Key Laboratory for Improving Quality and Productivity of Arable Land of Yunnan Province, Kunming 650201, China

**Keywords:** disease, high-throughput sequencing, pathogen identification, *Bacillus* spp.

## Abstract

The undefined microbial ecology of *Aconitum carmichaelii* root rot in western Yunnan constrains the advancement of eco-friendly control strategies. The identification of potential pathogenic determinants affecting *A. carmichaelii* growth is imperative for sustainable cultivation and ecosystem integrity. High-throughput sequencing was employed to profile microbial communities across four critical niches, namely rhizosphere soil, tuberous root epidermis, root endosphere, and fibrous roots of healthy and diseased *A. carmichaelii*. The physicochemical properties of corresponding rhizosphere soils were concurrently analyzed. Putative pathogens were isolated from diseased rhizospheres and tubers through culturing with Koch’s postulates validation, while beneficial microorganisms exhibiting antagonism against pathogens and plant growth-promoting (PGP) traits were isolated from healthy rhizospheres. Highly virulent strains (2F14, FZ1, L23) and their consortia were targeted for suppression. Strain DX3, demonstrating optimal PGP and antagonistic capacity in vitro, was selected for pot trials evaluating growth enhancement and disease control efficacy. Significant disparities in rhizosphere soil properties and bacterial/fungal community structures were evident between healthy and diseased cohorts. Fifteen putative pathogens spanning eight species across four genera were isolated: *Fusarium solani*, *F. avenaceum*, *Clonostachys rosea*, *Mucor racemosus*, *M. irregularis*, *M. hiemalis*, *Serratia liquefaciens*, and *S. marcescens*. Concurrently, eight PGP biocontrol strains were identified: *Bacillus amyloliquefaciens*, *B. velezensis*, *B. subtilis*, *B. pumilus*, and *Paenibacillus polymyxa.* Pot trials revealed that *Bacillus* spp. enhanced soil physiochemical properties through nitrogen fixation, phosphate solubilization, potassium mobilization, siderophore production, and cellulose degradation, significantly promoting plant growth. Critically, DX3 inoculation elevated defense-related enzyme activities in *A. carmichaelii*, enhanced host resistance to root rot, and achieved >50% disease suppression efficacy. This work delineates key pathogenic determinants of Yunnan *A. carmichaelii* root rot and identifies promising multifunctional microbial resources with dual PGP and biocontrol attributes. Our findings provide novel insights into rhizosphere microbiome-mediated plant health and establish a paradigm for sustainable disease management.

## 1. Introduction

*Aconitum carmichaelii* Debx. (Ranunculaceae), processed from daughter roots of *Aconitum carmichaelii*, is a renowned medicinal plant extensively distributed across Asia, notably in China, Korea, Japan, and India [1]. It serves as an essential ingredient in numerous traditional Chinese medicine prescriptions, and its major active substances are alkaloids, which exhibit a wide range of biological and pharmacological effects. It is widely prescribed for managing acute myocardial infarction, coronary heart disease, chronic heart failure, and rheumatic arthralgia [2]. During the pandemic of COVID-19, *A. carmichaelii* has been suggested as one of the key traditional Chinese medicinal herbs for the treatment of critical patients by the National Health Commission of China.

However, driven by its remarkable clinical efficacy and substantial demand in Traditional Chinese Medicine (TCM), *A. carmichaelii* cultivation has expanded considerably in China. Originally concentrated in historical production zones (e.g., Hanzhong, Shaanxi, and Jiangyou, Sichuan), its cultivation has now rapidly extended to Northwestern Yunnan [3]. Paradoxically, continuous monocropping of this herb has severely degraded soil quality, while suboptimal agricultural practices aimed at maximizing economic returns have exacerbated continuous cropping obstacles and disease outbreaks [4]. Continuous cropping obstacles (CCOs) represent a pervasive challenge in the cultivation of medicinal plants, particularly in root- and tuber-based species such as *A. carmichaelii*, as well as *Panax ginseng* and other plants from the Araliaceae family [5,6,7]. Due to their slow growth and long cultivation cycles, continuous monoculture often leads to depletion of soil nutrients, alterations in microbial communities, and increased susceptibility to diseases and pests, ultimately resulting in reduced yield and quality. Mechanistically, CCOs arise from altered soil physicochemical properties, autotoxic allelopathy, and pathogen-enriched microbiome shifts [8]. Specifically in *A. carmichaelii*, major pathogens—including *Fusarium oxysporum*, *Fusarium solani*, and *Sclerotium rolfsii*—induce root rot, southern blight (*S. rolfsii*), soft rot, and downy mildew, with synergistic coinfections frequently causing yield losses exceeding 50% [9,10]. Current reliance on chemical pesticides for disease management accelerates the development of pathogen resistance while generating substantial environmental pollution [11]. Numerous studies have demonstrated that biological control is an environmentally friendly approach capable of providing preventive and long-term, sustainable management effects. As a result, it is regarded as a suitable and sustainable alternative strategy.

Numerous studies have established that environmental conditions, including temperature, pH, and nutrient availability, exert significant influences on both plant growth and the pathogenicity of pathogenic microorganisms [12,13]. Concurrently, continuous cropping can induce alterations in the structure of rhizosphere soil microbial communities and soil chemical properties. The concomitant decline in beneficial microbes and proliferation of pathogenic microorganisms are hypothesized to be key factors contributing to reduced crop yield and increased disease incidence [14,15]. However, current research on crop diseases often adheres to the “single pathogen hypothesis” during pathogen identification, thereby overlooking the complex interactions within microbial communities. Investigations into *A. carmichaelii* root diseases have predominantly focused on southern blight caused by *S. rolfsii* and root rot associated with *F. solani* and *F. oxysporum* [16]. Root rot of *A. carmichaelii* caused by *Ilyonectria robusta* has also been reported in China [17]. Furthermore, explorations of potential pathogens in *A. carmichaelii* root diseases have largely concentrated on analyzing soil fungal diversity [18]. Notably, two bacterial strains (*Pseudomonas aeruginosa* and *Serratia marcescens*) were isolated from decaying post-harvest *A. carmichaelii* tubers and confirmed to be pathogenic, marking the first report of post-harvest decay in *A. carmichaelii* caused by these bacteria [3]. Recently, soft rot of *A. carmichaelii* caused by *Pectobacterium brasiliense* has also been documented in China [19]. While increasing research has identified various pathogens responsible for *A. carmichaelii* root diseases, studies simultaneously analyzing both bacterial and fungal microbiomes associated with the root system to explore the etiology of *A. carmichaelii* root rot remain scarce. The pathogenesis of *A. carmichaelii* root diseases is likely intricately linked to the complex microbial ecology and soil physicochemical factors. Therefore, a comprehensive analysis of potential pathogenic determinants, coupled with prospecting for beneficial antagonistic microorganisms, is imperative.

Soil-borne pathogens are well-established inducers of plant disease, while non-pathogenic microorganisms can benefit plants by enhancing nutrient use efficiency or suppressing pathogen infection [20]. It is widely recognized that rhizosphere microbes play a pivotal role in disease suppression and in producing phytohormones and compounds that directly influence plant health [21]. Consequently, prospecting for beneficial microbes to achieve biological control of soil-borne diseases has proven to be a viable strategy [22]. Among these beneficial microbes, the genus *Bacillus* is a dominant component of healthy plant microbiomes, renowned for its plant PGP and biocontrol activities. For instance, the *A. carmichaelii* endophyte *B. subtilis* JY-7-2L produces PGP agents such as β-1,3-glucanase, cellulase, protease, indole-3-acetic acid (IAA), and siderophores, promoting *A. carmichaelii* growth and effectively controlling root disease caused by *S. rolfsii* [16]. Similarly, *B. subtilis* HSY21 significantly reduces root rot in soybean caused by *F. oxysporum* and suppresses the expression of genes associated with *Fusarium* pathogenicity [23]. *Bacillus* strains exhibit remarkable stress tolerance and the ability to colonize plant surfaces. Consequently, the utilization of *Bacillus* spp. for plant disease management has become a major focus in biocontrol research [24]. Furthermore, the influence of soil physicochemical factors on root rot development cannot be overlooked. Studies collectively demonstrate that the occurrence and severity of plant root rot are influenced by climatic conditions and soil physicochemical properties at the planting site. For example, soil pH and total potassium content show significant correlations with the incidence of *Torreya grandis* root rot [25]. Therefore, an integrated analysis incorporating both microbial community diversity and soil physicochemical properties is essential for elucidating the etiology of *A. carmichaelii* diseases and developing effective control strategies.

The technological advantages of microbiomics enable the elucidation of complex host–microbe–environment interaction networks and the identification of key pathogenic and probiotic taxa. The pathogenesis of *A. carmichaelii* root rot may be associated with the enrichment of specific pathogens and a concomitant depletion of beneficial microbial consortia, whereby rhizosphere probiotics can enhance host resistance through ecological modulation. Previous research on *A. carmichaelii* root diseases has predominantly focused on analyzing fungal diversity within the soil [18,26], often concentrating on a single compartment (e.g., rhizosphere soil) or operating under a limited pathogen hypothesis. To address these limitations, this study employed high-throughput sequencing to comprehensively analyze bacterial and fungal microbiome diversity across multiple *A. carmichaelii* compartments, namely rhizosphere soil, fibrous roots, and tubers of both healthy and diseased plants. Concurrently, the physicochemical properties of the corresponding soils were characterized. This integrated approach facilitated the identification of key pathogenic taxa, whose virulence was subsequently validated. Building upon these findings, rhizobacteria exhibiting dual functions in promoting *A. carmichaelii* growth and disease suppression were screened. Furthermore, pot trials were conducted to evaluate whether the selected biocontrol strains could achieve synergistic effects by modulating soil physicochemical properties and enhancing plant defense enzyme activity. The overarching objectives of this study are to characterize the microbiome signatures across distinct *A. carmichaelii* compartments and to develop sustainable green control strategies that leverage functional microorganisms.

## 2. Materials and Methods

### 2.1. Sample Collection and Grouping

**Figure 1:**
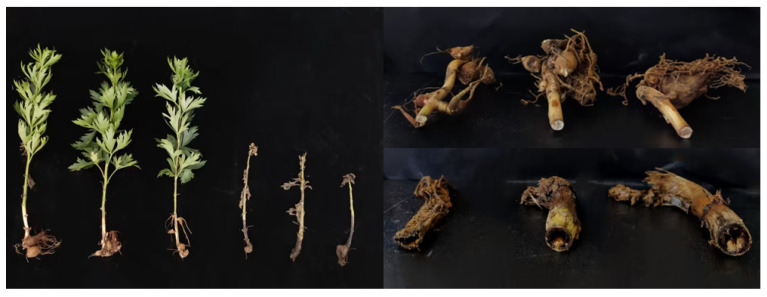
Phenotypic comparison of healthy and root rot-diseased *A. carmichaelii* plants.

The study site was located at the *A. carmichaelii* cultivation base in Jiguowowozi, Hutiaoxia Town, Shangri-La City, Diqing Tibetan Autonomous Prefecture, Yunnan Province, China (Coordinates: 27°30′ N, 99°54′ E). Sampling was conducted within a newly reclaimed planting area following a fallow period. *A. carmichaelii* plants in this region are typically planted between November and December of the preceding year. During the second consecutive year of cultivation, root rot symptoms emerged in mid-growth stage (April–June) across distinct zones within the field. Initial disease symptoms manifested as foliar yellowing, then progressing to root decay and eventual wilting of the aerial parts as the infection advanced (Figure 1). *A. carmichaelii* samples were collected in June 2023. Fifteen healthy plants and fifteen naturally infected plants were randomly selected. Soil surrounding the target plant (within a 30 cm diameter) was carefully removed. The soil monolith encompassing the *A. carmichaelii* tuber and root system was then carefully excavated to ensure intact retrieval of the entire plant. The collected plants were sequentially labeled as samples 1 through 30. Healthy and diseased *A. carmichaelii* samples were categorized as follows:

Rhizosphere soil samples were collected using the root-shaking method. The *A. carmichaelii* plants were subjected to moderate shaking to dislodge loosely adhering soil surrounding the root system. This rhizosphere soil was carefully collected using a sterile brush and transferred into sterile sample bags. Rhizosphere soil from every five *A. carmichaelii* plants was pooled to form one composite sample. Three replicate composite samples were prepared for both healthy and infected plant groups and were labeled as HT-1, HT-2, HT-3 (Healthy) and IT-1, IT-2, IT-3 (Infected), respectively. The collected rhizosphere soil samples were sieved through a 0.84 mm mesh and immediately placed in a 4 °C cooler for transport to the laboratory. Upon arrival, the sieved soil was divided into two aliquots, one stored at −80 °C for subsequent DNA extraction and the other stored at 4 °C for analysis of soil physicochemical properties.

Fibrous roots and tuber tissues were excised from *A. carmichaelii* plants using sterile scissors. These tissues were subjected to elution with phosphate-buffered saline (PBS) and subsequently immersed in sterile PBS for ultrasonic treatment (90 W, 30 s on/30 s off cycles) for 5 min. Fibrous root tissue from every five *A. carmichaelii* plants was pooled to form one composite sample. Composite samples from healthy and infected plants were labeled as HG-1, HG-2, HG-3 (Healthy Groups) and IG-1, IG-2, IG-3 (Infected Groups), respectively. Tuber epidermis was aseptically collected using a surface-sterilized scalpel. Epidermal tissue from every five *A. carmichaelii* tubers was pooled to form one composite sample. Composite samples from healthy and infected tubers were labeled as HB-1, HB-2, HB-3 (Healthy Bark) and IB-1, IB-2, IB-3 (Infected Bark), respectively. Following epidermis removal, internal tuber tissue was aseptically collected using a sterile scalpel. Internal tissue from every five *A. carmichaelii* tubers was pooled to form one composite sample. Composite samples from healthy and infected tubers were labeled as HN-1, HN-2, HN-3 (Healthy Nucleus) and IN-1, IN-2, IN-3 (Infected Nucleus), respectively. All collected tissue samples (fibrous roots, epidermis, and internal tissue) were stored at −80 °C for subsequent DNA extraction.

### 2.2. Analysis of Soil Physicochemical Properties

Soil pH was measured using a PHS-3E pH meter (INASE Scientific Instrument, Shanghai, China). Organic matter (OM) content was quantified via the potassium dichromate volumetric method. Total nitrogen (TN) content was measured using an elemental analyzer. Total phosphorus (TP) and available phosphorus (AP) contents were assessed by molybdenum–antimony anti-spectrophotometry. Total potassium (TK) and available potassium (AK) contents were determined by flame photometry. Alkaline hydrolyzable nitrogen (AN) content was analyzed using the alkali-hydrolyzable diffusion method.

### 2.3. Soil Genomic DNA Extraction and PCR Amplification

Soil microbial DNA was extracted using the E.Z.N.A.^®^ Soil DNA Kit (Omega Bio-tek, Norcross, GA, USA), and plant tissue DNA was extracted using the MP Fastprep-24 5G system (MP Biomedicals, Irvine, CA, USA). The integrity of genomic DNA was then verified by 1% agarose gel electrophoresis. The bacterial primers in reference [27] were used to amplify the V3–V4 region of the 16S rRNA gene. The fungal primers in reference [26] were used to amplify the ITS1 region of the ITS gene. ASVs aligned to chloroplast or mitochondrial sequences were removed from the study. PCR amplification was carried out using TransStart FastPfu DNA Polymerase (TransGen Biotech, Beijing, China) with a reaction system containing the following: 4 μL of 5× FastPfu Buffer, 2 μL of 2.5 mM dNTPs, 0.8 μL of each primer (5 μM), 0.4 μL of FastPfu Polymerase, and 10 ng of template DNA, supplemented with ddH_2_O to a final volume of 20 μL. The PCR cycling conditions consisted of an initial denaturation at 95 °C for 5 min; 25 cycles of denaturation at 95 °C for 30 s, annealing at 55 °C for 30 s, and extension at 72 °C for 45 s; followed by a final extension at 72 °C for 10 min. Each sample was amplified in three independent PCR replicates. Amplicons were excised from 2% agarose gels and purified using the AxyPrep DNA Gel Extraction Kit (Axygen Biosciences, Union City, CA, USA). The purified products were examined by 2% agarose gel electrophoresis and quantified using a Qubit^®^ 4.0 Fluorometer (Thermo Fisher Scientific, Waltham, MA, USA). Purified PCR products from soil and tissue samples were subjected to paired-end sequencing on the Illumina NovaSeq 6000 PE250 platform by Shanghai Majorbio Bio-Pharm Technology (Majorbio, Shanghai, China), generating both forward and reverse reads.

Raw sequences obtained from high-throughput sequencing were analyzed on the I-Sanger Cloud Platform (www.i-sanger.com) using QIIME2 (v2022.2) and the UNITE 8.0/ITS_fungi and SILVA138/16S_bacteria database. After processing including quality control (fastp v0.19.6), denoising (DADA2), merging (FLASH v1.2.7), and chimera removal, a total of 2,005,483 high-quality bacterial sequences and 2,586,465 high-quality fungal sequences were obtained. ASV clustering and taxonomic identification were performed at a 97% similarity threshold. Following rarefaction based on the minimum sample sequencing depth, 40,762 bacterial ASVs and 9924 fungal ASVs were retained, with an average coverage of 99.4%.

### 2.4. Isolation and Identification of Root Rot Pathogens from A. carmichaelii

Fresh diseased *A. carmichaelii* tubers and associated rhizosphere soil were collected for pathogen isolation. White mycelia observed on tuber surfaces were transferred to Potato Dextrose Agar (PDA, 4.0 g/L Potato dextrose broth powder, 20.0 g/L Glucose, 15.0 g/L Agar) plates using sterile toothpicks. Diseased tissues and soil were homogenized in sterile phosphate-buffered saline (PBS, 8.0 g/L NaCl, 0.2 g/L KCl, 1.44 g/L Na_2_HPO_4_, 0.24 g/L KH_2_PO_4_), serially diluted, and plated on PDA (for fungi) and Luria–Bertani (LB, 10.0 g/L Tryptone, 5.0 g/L Yeast extract, 10.0 g/L NaCl, 15.0 g/L Agar) agar (for bacteria). All plates were incubated and inverted at 30 °C for 7 days. Distinct fungal colonies were purified by subculturing hyphal tips (harvested with 8 mm sterile cork borers) onto fresh PDA. Bacterial colonies were streak-purified on LB agar using sterile loops.

Molecular identification was as follows: For fungal isolates, the primers from reference [28] were used in 50 μL reactions to amplify the ITS region. The reaction mixture contained 25 μL 2× PCR Master Mix (Vazyme Biotech, Nanjing, China), 2 μL of each primer (10 μM), 2 μL template DNA (50 ng/μL), and 19 μL nuclease-free water. Cycling conditions: 94 °C for 5 min; 30 cycles of 94 °C (30 s), 55 °C (30 s), and 72 °C (1 min); and final extension of 72 °C for 10 min. Bacterial isolates: The primers from reference [16] were used to amplify the 16S rRNA gene in 25 μL reactions containing of 12.5 μL 2× Phanta Max Master Mix (Vazyme Biotech, Nanjing, China), 1 μL of each primer (10 μM), 0.5 μL bacterial suspension (OD_600_ 0.6−0.8), and 10 μL nuclease-free water. Cycling conditions: 95 °C for 5 min; 35 cycles of 95 °C (30 s), 60 °C (30 s), and 72 °C (2 min); and final extension of 72 °C for 5 min. Amplicons were electrophoresed on 1.5% agarose gels to confirm target sizes. Validated PCR products were sequenced by Kunming Tsingke Biotechnology (Kunming, China).

### 2.5. Pathogenicity Assay of Putative Pathogens

Healthy fresh tubers of *A. carmichaelii* were surface-sterilized by sequential washing with running tap water, 75% ethanol for 5 min, and three rinses in sterile distilled water, each rinse was performed for 30 s. After air-drying, tubers were aseptically sectioned into 8 mm thick slices using sterile scalpels in a laminar flow cabinet. Slice surfaces were wounded with sterile needles. Fungal inoculation: Mycelial plugs (6 mm diameter) from pure cultures of putative fungal pathogens grown on PDA were placed onto wound sites. Controls received sterile PDA plugs. Each treatment included three replicate slices. Bacterial inoculation: Bacterial suspensions (OD_600_ = 0.8) of putative pathogens in LB broth were prepared. Aliquots (50 μL) were pipetted onto wound sites. Controls received sterile LB broth. Each treatment included three replicate slices. Positive controls: Reference strains of *F. solani* and *F. oxysporum* (previously confirmed as pathogenic, was kindly provided by Professor Yueqiu He from Yunnan Agricultural University [29,30]) were inoculated using identical protocols. All strain inoculations and controls were performed in parallel groups. Inoculated slices were maintained in a growth chamber (28 °C, 70% relative humidity). Symptom development was monitored daily. Infection was confirmed when lesion extension exceeded 2 mm beyond the inoculation site. Pathogens were re-isolated from symptomatic tissues onto PDA/LB media and re-identified morphologically/molecularly.

### 2.6. Isolation and Functional Characterization of Rhizosphere Beneficial Microbes

#### 2.6.1. Microbial Isolation and Preservation

Fresh rhizosphere soil from healthy *A. carmichaelii* plants was processed within 24 h. A 10 g soil aliquot was suspended in 90 mL of sterile phosphate-buffered saline (PBS, pH 7.2) and homogenized at 150 rpm (25 °C, 30 min). After sedimentation (10 min), serial dilutions (10^−1^, 10^−2^, 10^−3^, 10^−4^, and 10^−5^) were prepared. Aliquots (100 μL) of each dilution were spread-plated on LB agar and incubated at 28 °C for 3–5 days. Morphologically distinct colonies were streak-purified and cryopreserved at −80 °C in 30% (*v*/*v*) glycerol for subsequent analysis.

#### 2.6.2. Molecular Identification

Bacterial species identification followed the 16S rRNA gene amplification protocol detailed in Section 2.4.

#### 2.6.3. PGP Trait Screening

Isolates were evaluated for six PGP traits using established methodologies. IAA production: quantified colorimetrically using Salkowski reagent [31]. Phosphate solubilization: Pikovskaya’s (PVK) agar plate assay. Potassium solubilization: Aleksandrow agar plate assay [32]. Siderophore production: chrome azurol S (CAS) agar plate assay [33]. Nitrogen fixation: growth on Ashby’s nitrogen-free agar (Ashby) [34]. Cellulase activity: carboxymethylcellulose (CMC-Na) plate assay [35]. Liquid assay: bacterial cultures of each strain (OD_600_ = 0.8) were inoculated at 1% (*v*/*v*) into 50 mL of PVK liquid medium with Ca_3_(PO_4_)_2_ and lecithin as phosphorus sources, and into Aleksandrov liquid medium containing potassium feldspar powder [32]. Three types (using Ca_3_(PO_4_)_2_, lecithin, and potassium feldspar powder as functional media) of uninoculated liquid media served as blank controls. All treatments were adjusted to pH 7.0 and performed in triplicate. Cultures were incubated at 28 °C with shaking at 150 r/min for 7 days. After cultivation, the broth of each strain in the PVK liquid medium and Aleksandrov liquid medium was centrifuged at 10,000 r/min for 5 min, and the supernatant was collected for further analysis, with the supernatant from blank media used as the control. The amount of dissolved phosphate was determined using the molybdenum blue method [36], and the dissolved potassium content was measured by flame photometry [37]. The pH of the culture broth after inoculation of each strain in the different phosphorus/potassium source media was measured using a PHS-3E pH meter.

#### 2.6.4. Antagonistic Activity Assay

The antagonistic effects of the eight selected PGP bacterial strains against eight target pathogens were assessed using a dual culture plate assay. The target pathogens included those previously confirmed to be pathogenic to aconite through pathogenicity tests, as well as *Sclerotium rolfsii*, which causes southern blight in *A. carmichaelii*. On PDA medium (9 cm plates), each pathogen was inoculated centrally with a mycelial plug, placed with the mycelial side facing downward. For the treatment groups, purified single colonies of the same bacterial strain were inoculated at four positions 2.5 cm above, below, left, and right from the central pathogen. The control groups received no bacterial colonies. All treatments were performed in triplicate under parallel experimental conditions. All plates were incubated at 28 °C for 7 days. The inhibition rate (%) was calculated as

Inhibition rate (%) = (*R* − *r*)/*R*

where *R* and *r* are the mycelia radial growth on the control fungal plate and mycelia radial growth on the antagonistic strain-treated plate, respectively.

Additionally, bacterial isolates were spot-inoculated on LB agar blended with broth cultures of potential pathogenic bacteria (OD_600_ = 1.0, 2% *v*/*v*) to verify antagonistic capacity. Results were interpreted based on presence/absence of clear inhibition zones.

### 2.7. Pot Experiment for Elite Strain Validation

#### 2.7.1. Experimental Setup

Trials were conducted in a greenhouse at Yunnan Agricultural University (25°08′ N, 102°45′ E) using field soil with the following properties: pH 7.37, AN 114.13 mg/Kg, AP 17.04 mg/Kg, AK 243.61 mg/Kg, and OM 35.79 g/Kg. Pathogens *Fusarium* spp. 2F14, *Mucor* spp. FZ1, and *Serratia marcescens* L23 (validated in Section 2.5) were selected as inoculation targets. *B. amyloliquefaciens* DX3 was chosen based on superior in vitro plant growth-promoting and antagonistic activities (Section 2.6).

#### 2.7.2. PGP Effect Assessment

Eight bacterial strains (C77, C31, D11, DX3, DX37, E1, X1, and Y8) were cultured in LB broth at 30 °C and 160 rpm for 72 h. Cells were harvested by centrifugation (8000× *g*, 10 min), washed twice with sterile water, and resuspended to 10^9^ CFU/mL. *A. carmichaelii* seedlings were root-drenched with 10 mL suspension per plant at 15-day intervals. Controls received sterile water. Each pot contained three *A. carmichaelii* plants, with three replicates per treatment. The pots were arranged randomly and periodically repositioned. During plant growth, watering was applied as needed, and no fertility agents other than the bacterial suspension application were introduced. Upon maturation of the *A. carmichaelii* plants, the following parameters were measured: plant height, stem diameter, number of leaves, chlorophyll content, biomass, as well as rhizosphere soil pH, available N/P/K, and OM. For soil physiochemical properties analysis, three samples were collected per treatment and determined according to the methods described in Section 3.2. Agronomic traits of the plants were assessed on a per-pot basis, with the mean values of parameters from three plants per treatment used as representative data for each treatment group.

#### 2.7.3. Biocontrol Efficacy Evaluation

Inoculum preparation: DX3—LB culture adjusted to 10^9^ CFU/mL. Bacterial pathogen (L23)—LB culture adjusted to 10^9^ CFU/mL. Fungal pathogens (FZ1 and 2F14)—PDA broth filtrate adjusted to 10^7^ conidia/mL. The experimental design was as follows:

Control group (CK): LB liquid medium without inoculation; Negative control: T1 (inoculated with biocontrol strain), DX3; Positive control: T2 (inoculated with pathogens), L23, 2F14, FZ1, L23 + 2F14, L23 + FZ1, 2F14 + FZ1, L23 + 2F14 + FZ1; Treatment group: T3 (inoculated with biocontrol strain + pathogens), DX3 + L23, DX3 + 2F14, DX3 + FZ1, DX3 + L23 + 2F14, DX3 + L23 + FZ1, DX3 + 2F14 + FZ1, DX3 + L23 + 2F14 + FZ1. Each pot contained three *A. carmichaelii* plants, with six replicates per treatment and three parallel groups. During the vigorous growth period of *A. carmichaelii* (April), the fermentation culture of strain DX3 (10 mL/plant) was inoculated to the roots of *A. carmichaelii* in T1 and T3 treatments. Two days later, pathogen filtrates were inoculated to T2 and T3 treatments, with an inoculation amount of 10 mL/plant for L23 and 1 × 10^7^ CFU/mL for 2F14 and FZ1. During plant growth, soil moisture was monitored using a soil moisture data logger and maintained at 60–80% of field capacity. The inoculations of the biocontrol strain and pathogens were performed every half month until harvest at the end of May.

#### 2.7.4. Disease Assessment and Physiological Analysis

Disease severity index (DSI):

Tubers were scored using a 0–5 scale as follows: 0—no symptoms; 1—surface rot ≤ 10% or internal rot 5–10%; 2—internal rot 10–40%, no lateral root rot; 3—internal rot 40–50% with lateral root rot; 4—internal rot 50–70% with root abscission; 5—internal rot > 70% or complete decay.

DSI (%) = [Σ (Number of plants × Grade)/(Total plants × 5)] × 100(1)

Biocontrol efficacy (BE):

BE (%) = (DSI_T2 − DSI_T3)/DSI_T2 × 100(2)

Physiological assays: For each treatment, three plants were randomly selected to form one sample, and leaves from the same position of the *A. carmichaelii* plants were collected each time for determining peroxidase (POD) activity, polyphenol oxidase (PPO) activity, and phenylalanine ammonia-lyase (PAL) activity, with three replicates measured per treatment. The activity of PPO was determined using kits (Boxbio, Beijing, China), in combination with the method described in reference [38]. The activities of PAL and POD were determined according to the method described in reference [39] and the instructions of the assay kit.

### 2.8. Statistical and Bioinformatics Analyses

Data processing was performed using IBM SPSS Statistics 25 for statistical analysis, and Origin 2021 software for figure generation. One-way analysis of variance (ANOVA) with Tukey’s HSD test for significant differences was applied for comparisons between treatments, along with tests for homogeneity of variances. Sequencing sequences of strains were assembled using DNAMAN 7.0, aligned via BLAST (https://blast.ncbi.nlm.nih.gov/Blast.cgi, accessed on 6 May 2025), and phylogenetic trees were constructed using the maximum likelihood method in MEGA 6.0 software (with 1000 bootstrap replications). Microbial diversity data were analyzed on the I-Sanger Cloud Platform (www.i-sanger.com). Group differences in alpha diversity indices were evaluated using the Kruskal–Wallis test with FDR correction for multiple comparisons in R-3.3.1 (stat package). Principal coordinate analysis (PCoA) was conducted in R-3.3.1 with the vegan package at the microbial genus level, and intergroup differences were assessed using the Adonis test. Mantel tests were performed based on Bray–Curtis distance matrices, with Pearson’s method used for correlation coefficients.

## 3. Results

### 3.1. Rhizosphere Soil Properties of Healthy vs. Diseased A. carmichaelii

As detailed in Table 1, significant differences (*p* < 0.05) were observed in rhizosphere soil properties between healthy and diseased plants. Compared to healthy plants, diseased specimens exhibited the following: decreases in pH (−17.12%) and available nitrogen (AN, −7.94%), and increases in total nitrogen (TN, +7.48%), total phosphorus (TP, +15.12%), total potassium (TK, +14.29%), available phosphorus (AP, +66.07%), available potassium (AK, +307.64%), and organic matter (OM, +19.35%).

### 3.2. Microbial Diversity and Community Structure

**Figure 2:**
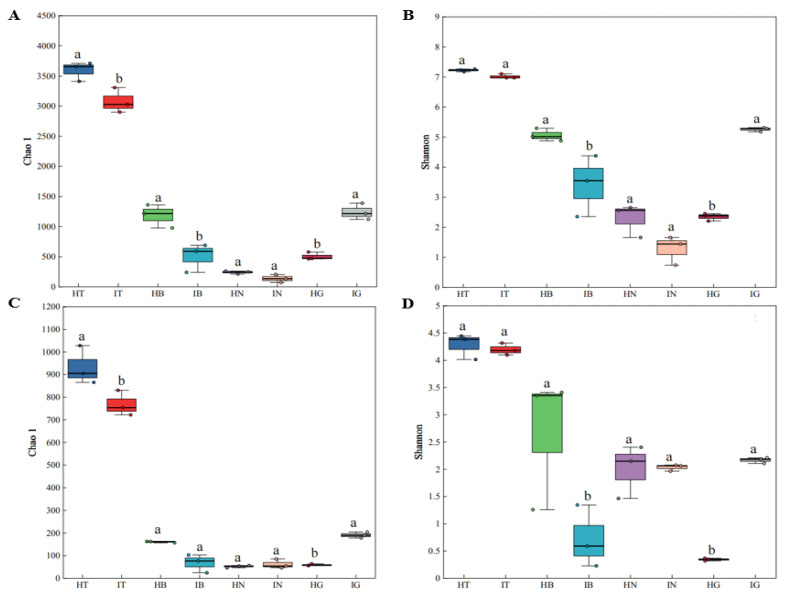

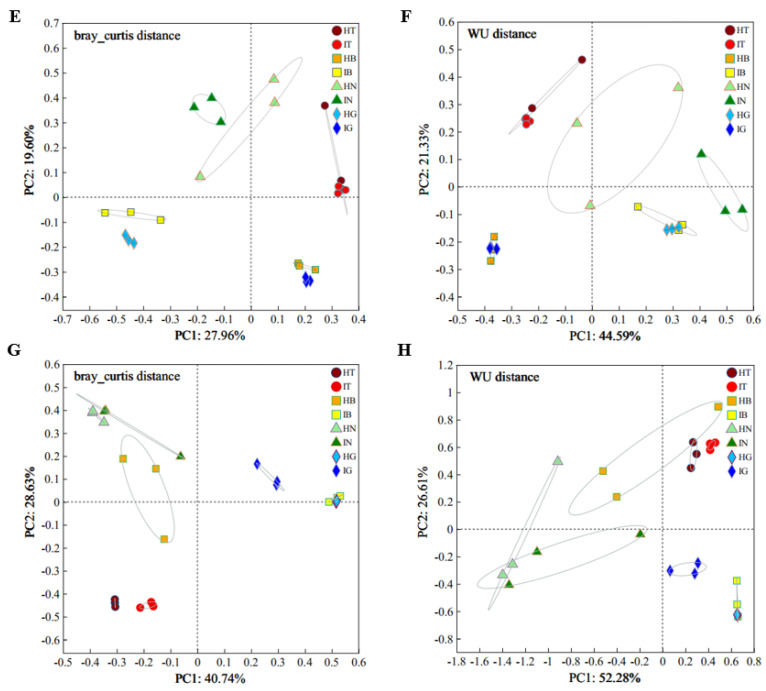
Alpha and beta diversity of microbial communities. (**A**–**D**) Alpha diversity indices: (**A**) bacterial Chao1, (**B**) bacterial Shannon, (**C**) fungal Chao1, and (**D**) fungal Shannon. Significant differences between the same tissues of healthy and diseased *A. carmichaelii* are denoted by different lowercase letters (*p* < 0.05). (**E**–**H**) Beta diversity of bacterial communities based on Bray–Curtis ((**E**), R = 0.8274, *p* = 0.001) and weighted UniFrac (WU) ((**F**), R = 0.8558, *p* = 0.001) distances; Beta diversity of fungal communities based on Bray–Curtis ((**G**), R = 0.79, *p* = 0.001) and WU ((**H**), R = 0.7192, *p* = 0.001) distances.

Alpha diversity analysis (Chao1, Shannon indices) of fibrous roots, root tuber epidermis, root tuber interior, and rhizosphere soil in healthy and diseased *A. carmichaelii* (Figure 2A–D) revealed the following: Rhizospheric Chao1 and Shannon indices were significantly higher than other compartments in both health states. Healthy rhizosphere exhibited significantly greater Chao1 but comparable Shannon indices versus diseased rhizosphere. Diseased fibrous roots showed significantly elevated Chao1 and Shannon indices relative to healthy fibrous roots. Epidermal and root tuber interior compartments showed no consistent differences, though healthy plants demonstrated numerically higher indices across these tissues. Beta diversity analysis using Bray–Curtis and WU distances (Figure 2E–H) indicated significant structural differences in microbial communities between compartments and health states. Some distinct clustering patterns were observed as follows: HT and IT were aggregated, as were HN and IN; HB vs. IB exhibited scattered distribution; and HG vs. IG showed divergent clustering.

### 3.3. Microbial Community Composition

**Figure 3:**
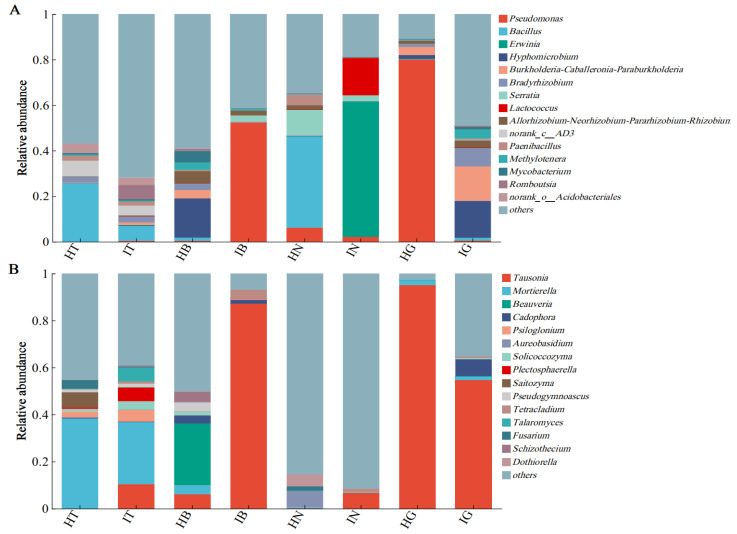
Relative abundance of bacterial (**A**) and fungal (**B**) communities at the genus level in the rhizosphere soil, root tuber epidermis, root tuber interior, and fibrous roots of healthy and diseased *A. carmichaelii*.

Analysis of bacterial community composition at the genus level in the rhizosphere, epidermis, root tuber interior, and fibrous roots of healthy and diseased *A. carmichaelii* plants (Figure 3) revealed distinct profiles. In healthy plants, the dominant bacterial genera were *Bacillus*, *Pseudomonas*, and *Hyphomicrobium*, while diseased plants were predominantly colonized by *Pseudomonas*, *Erwinia*, and *Hyphomicrobium*. Compared to healthy rhizosphere soil (HT), a significant reduction (18.84%) in the relative abundance of *Bacillus* was observed in infected rhizosphere soil (IT). Similarly, compared to healthy root tuber interior (HN), infected root tuber interior (IN) exhibited a substantial increase (59.34%) in *Erwinia* abundance alongside a significant decrease (40.02%) in *Bacillus*. Notably, *Pseudomonas* and *Hyphomicrobium* remained the primary bacterial genera in both healthy and diseased epidermal and fibrous root tissues (Figure 3A).

Fungal community analysis (Figure 3B) identified *Tausonia*, *Mortierella*, *Beauveria*, and *Aureobasidium* as the predominant genera in healthy plants; however, diseased plants harbored a distinct fungal consortium dominated by *Tausonia*, *Mortierella*, *Cadophora*, *Psiloglonium*, *Plectosphaerella*, and *Talaromyces*. A significant reduction (12.05%) in *Mortierella* abundance was detected in IT compared to HT. *Beauveria* was the dominant genus in healthy root tuber epidermis (HB), constituting 26.33% of the relative abundance, but was undetectable in infected root tuber epidermis (IB). *Tausonia* was highly abundant in both healthy and diseased epidermal and fibrous root tissues, with relative abundances of 87.16%, 95.13%, and 54.57% in IB, healthy fibrous roots (HG), and infected fibrous roots (IG), respectively (Figure 3B).

### 3.4. Differential Abundance Analysis

**Figure 4:**
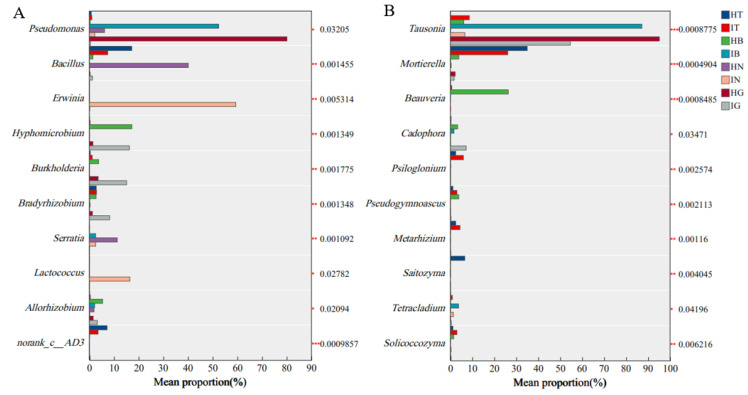
Differentially abundant bacterial (**A**) and fungal (**B**) taxa between healthy and diseased *A. carmichaelii*. *, **, *** denote significant differences at *p* < 0.05, *p* < 0.01, and *p* < 0.001, respectively, based on Kruskal–Wallis tests.

Analysis of genus-level differential abundance in bacterial communities associated with the rhizosphere, epidermis, root tuber interior, and fibrous roots of healthy versus diseased *A. carmichaelii* plants (Figure 4A) identified several key taxa exhibiting significant differences, including *Pseudomonas*, *Bacillus*, *Erwinia*, *Hyphomicrobium*, *Burkholderia*, *Bradyrhizobium*, *Serratia*, *Lactococcus*, and *Allorhizobium*. Notably, the relative abundance of *Bacillus* was significantly reduced in diseased plants. Conversely, *Erwinia*, *Burkholderia*, and *Bradyrhizobium* showed significantly increased relative abundances in diseased plants. Strikingly, *Erwinia* was exclusively detected in the root tuber interior of diseased plants, where it constituted 59.34% of the bacterial community. Similarly, fungal community analysis (Figure 4B) revealed significant differences in genera such as *Tausonia*, *Mortierella*, *Beauveria*, *Cadophora*, *Psiloglonium*, *Pseudogymnoascus*, *Metarhizium*, *Saitozyma*, *Tetracladium*, and *Solicoccozyma*. The relative abundances of *Mortierella* and *Beauveria* were significantly diminished in diseased plants. Furthermore, *Tausonia* exhibited significant differential abundance across various plant compartments in both healthy and diseased states. Based on these differential abundance patterns, it is hypothesized that *Erwinia* may function as a potential pathogen contributing to root disease in *A. carmichaelii*. Additionally, the onset of disease appears to be associated with significant alterations in the relative abundances of *Bacillus*, *Mortierella*, *Beauveria*, and *Tausonia*.

### 3.5. Correlations Between Soil Physicochemical Properties and A. carmichaelii Root Rot

**Figure 5:**
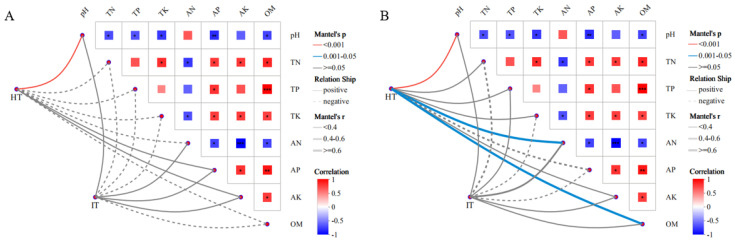
Correlation heatmaps between microbial communities and rhizosphere soil physicochemical properties in healthy (HT) and diseased (IT) *A. carmichaelii*. (**A**) Bacterial communities. (**B**) Fungal communities. HT: microbial community of healthy rhizosphere; IT: microbial community of infected rhizosphere. *, **, *** denote significant differences at 0.01 < *p* ≤ 0.05, 0.001 < *p* ≤ 0.01, and *p* ≤ 0.001, respectively.

Correlations between rhizosphere bacterial and fungal communities and soil physicochemical properties in healthy and diseased *A. carmichaelii* were assessed based on Bray–Curtis dissimilarity matrices. At the bacterial genus level, the rhizosphere microbial community of healthy plants exhibited a highly significant positive correlation (*p* < 0.01) with soil pH (Figure 5A). Similarly, at the fungal genus level, the rhizosphere microbial community of healthy plants showed a highly significant positive correlation (*p* < 0.01) with pH, along with significant positive correlations (*p* < 0.05) with available nitrogen (AN) and organic matter (OM) content (Figure 5B).

### 3.6. Pathogenicity Assay and Identification of Potential Pathogens

**Figure 6:**
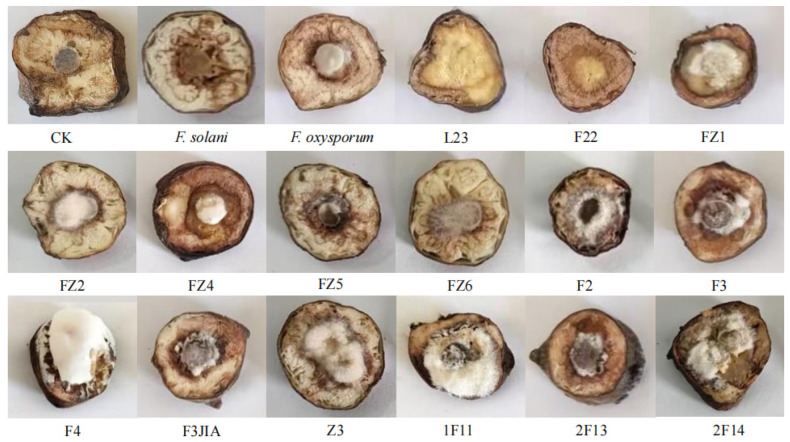
Pathogenicity validation of isolated potential pathogens toward *A. carmichaelii* root tubers.

The pathogenicity of the isolated potential pathogens was evaluated, leading to the identification of strains capable of inducing root tuber rot in *A. carmichaelii* (Figure 6). Following inoculation, decayed zones of varied sizes developed around the inoculation site on the root tubers. These symptoms closely resembled those caused by the positive controls *F. solani* and *F. oxysporum*. Notably, mycelial growth was observed on the surface of some inoculated tubers, eventually covering the decayed tissues. Inoculation with bacterial suspensions resulted in water-soaked decay with exudation. In contrast, control tubers exhibited no discernible disease symptoms. Quantitative assessment confirmed that 15 isolates induced distinct rot symptoms in *A. carmichaelii*, analogous to those produced by the positive controls. To fulfill Koch’s postulates, the putative pathogens were re-isolated from the decayed tuber tissues onto pure culture. Subsequent morphological characterization of these re-isolated strains confirmed characteristics identical to those of the original inocula.

**Figure 7:**
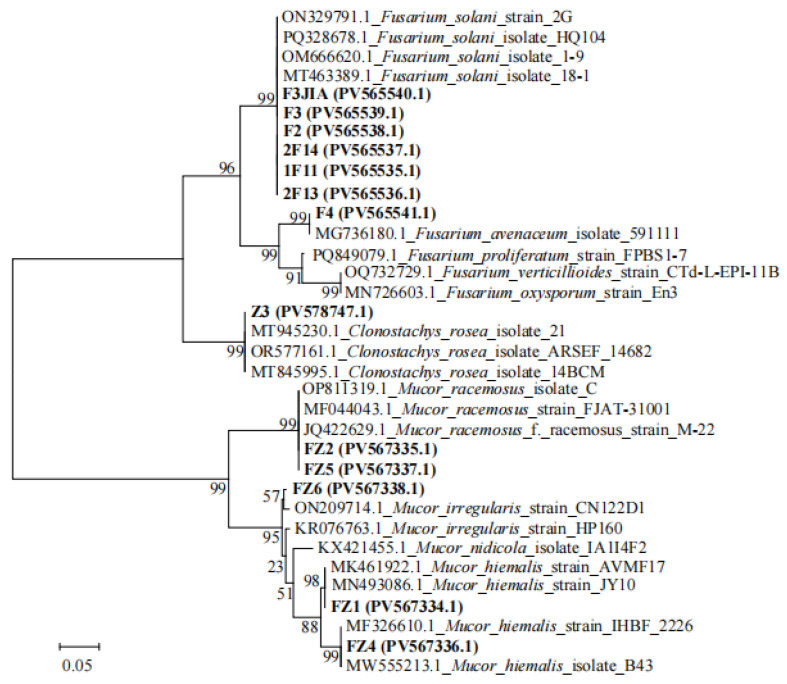
Maximum likelihood phylogenetic tree of potential fungal pathogens based on ITS gene sequences. Bootstrap support values are shown at branch nodes. GenBank accession numbers are indicated in parentheses. Scale bar = 0.05 nucleotide substitutions per site.

**Figure 8:**
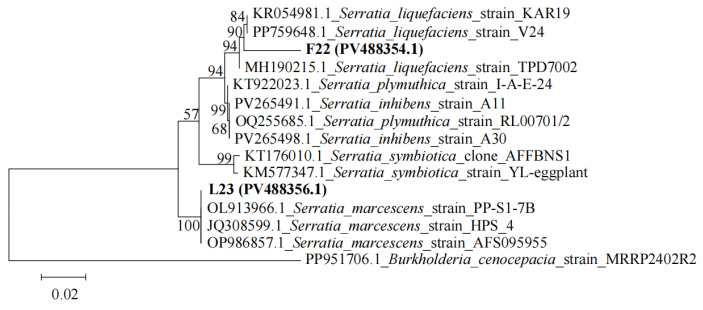
Maximum likelihood phylogenetic tree of potential bacterial pathogens based on 16S rRNA gene sequences. Bootstrap values are indicated at branch nodes. GenBank accession numbers are shown in parentheses. Scale bar = 0.02 nucleotide substitutions per site.

Phylogenetic analysis based on the ITS region (Figure 7) was employed to identify the isolated potential fungal pathogens. Isolates F3JIA, F2, F3, 1F11, 2F13, and 2F14 clustered closely with reference strains ON329791.1, PQ328678.1, OM666620.1, and MT463389.1 (all *F. solani*). Combined with morphological characteristics, these isolates were preliminary identification as *Fusarium solani*. Isolate F4 clustered closely with reference strain MG736180.1 (*F. avenaceum*). Combined with morphological characteristics, its preliminary identification was *Fusarium avenaceum*. Isolate Z3 exhibited close phylogenetic affinity to reference strains MT945230.1, OR577161.1, and MT845995.1 (all *C. rosea*). Morphological congruence led to its putative identification as *Clonostachys rosea*. Isolates FZ2 and FZ5 clustered with reference strains OP811319.1, MF044043.1, and JQ422629.1 (all *M. racemosus*). Based on combined molecular and morphological data, they were preliminarily identified as *Mucor racemosus*. Isolate FZ6 clustered closely with reference strain ON209714.1 (*M. irregularis*). Combined with morphological characteristics, its preliminary identification as *Mucor irregularis*. Isolate FZ1 grouped with reference strains including MN493086.1 (*M. hiemalis*), while isolate FZ4 clustered with strains such as MF326610.1 (*M. hiemalis*). Morphological features corroborated their preliminary identification as *Mucor hiemalis*. For potential bacterial pathogens, phylogenetic analysis utilizing the 16S rRNA gene was conducted (Figure 8). The isolated strain F22 showed close phylogenetic affinity to strains KP054981.1 and PP759648.1 (both *S. liquefaciens*) within the same genus. Based on morphological characteristics, it was preliminarily identified as *S*. *liquefaciens*. Isolate L23 exhibited close phylogenetic relatedness to reference strains OL913966.1, JQ308599.1, and OP986857.1 (all *S. marcescens*). Morphological evaluation confirmed its preliminary identification as *Serratia marcescens*. The GenBank accession numbers of all isolated strains are labeled on the phylogenetic tree. Among all isolated potential pathogens, strains L23 (*S. marcescens*), FZ1 (*M. hiemalis*), and 2F14 (*F. solani*) exhibited the most pronounced virulence in inducing *A. carmichaelii* tuber rot. Consequently, these three strains were selected as targets for subsequent biocontrol evaluation in pot experiments.

### 3.7. Plant Growth-Promoting Effects of Rhizosphere Beneficial Microorganisms

**Figure 9:**
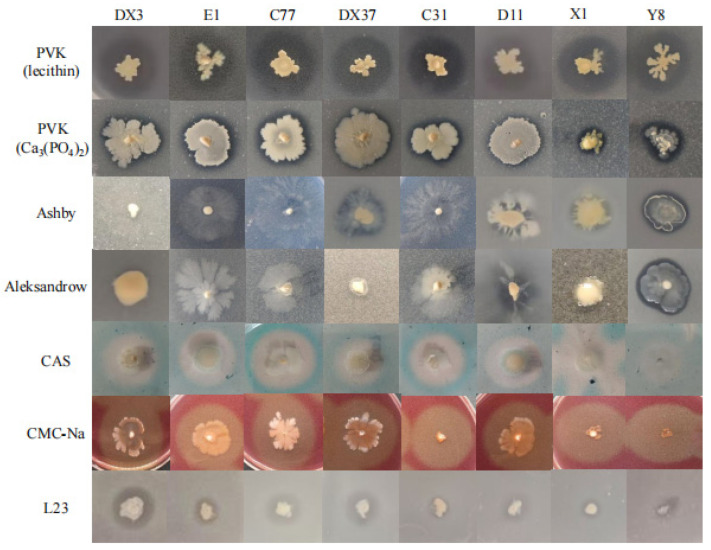
Functional traits of eight beneficial bacterial strains on specialized media and their antagonistic activity against strain L23.

**Figure 10:**
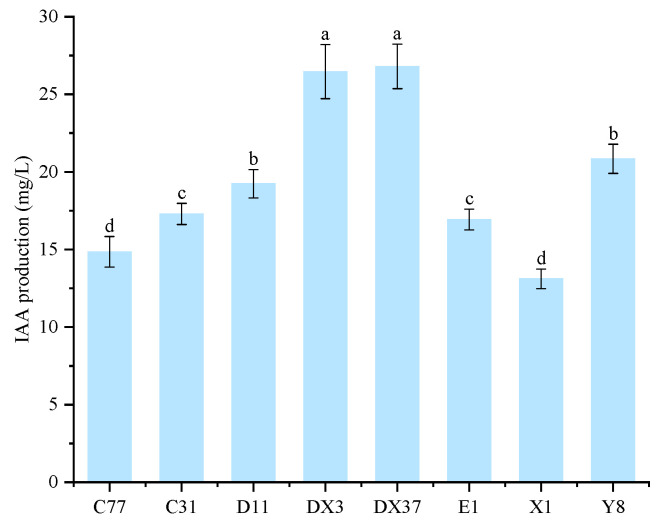
IAA production by eight beneficial bacterial strains. Bars labeled with different lowercase letters indicate statistically significant differences (Tukey’s HSD test, *p* < 0.05).

**Figure 11:**
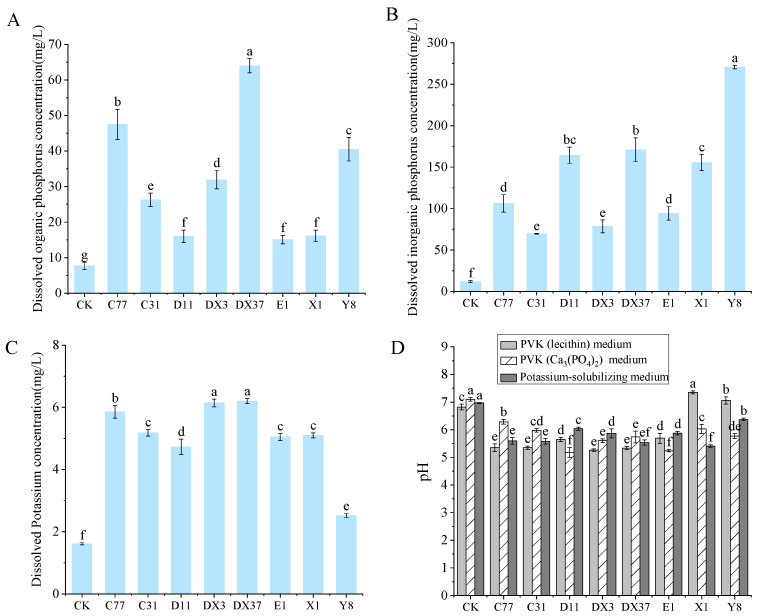
Phosphate solubilization, potassium dissolution, and broth pH dynamics by eight beneficial bacterial strains. (**A**) Organic phosphate solubilization. (**B**) Inorganic phosphate solubilization. (**C**) Potassium dissolution. (**D**) pH changes in culture broth. Bars labeled with different lowercase letters indicate statistically significant differences (Tukey’s HSD test, *p* < 0.05).

Beneficial microorganisms were isolated from the rhizosphere soil of healthy *A. carmichaelii* plants. Eight strains (DX3, E1, C77, DX37, C31, D11, X1, and Y8) demonstrating positive plant growth-promoting traits were selected based on their ability to produce IAA and siderophores and their capacity for phosphate solubilization (both organic and inorganic), potassium dissolution, nitrogen fixation, and cellulose degradation. Visualization assays on specific agar media revealed distinct halos (siderophore production and cellulose degradation), clear zones (phosphate solubilization), or growth (nitrogen fixation) associated with each PGP trait for these isolates (Figure 9). These results indicate the significant PGP potential of the selected strains. Quantitative analysis of IAA production (Figure 10) confirmed that all eight strains synthesized IAA, with concentrations ranging from 13.11 mg/L to 26.79 mg/L. Notably, strains DX3 and DX37 exhibited significantly higher (*p* < 0.05) IAA production (26.46 mg/L and 26.79 mg/L, respectively) compared to the other strains. The PGP capabilities were further quantified (Figure 11): The amount of solubilized organic phosphate varied significantly among strains, ranging from 15.10 mg/L to 63.98 mg/L. Strain DX37 demonstrated the highest solubilization capacity (63.98 mg/L), significantly exceeding (*p* < 0.05) the others. Solubilized inorganic phosphate levels ranged from 69.80 mg/L to 270.69 mg/L. Strain Y8 exhibited the most potent activity (270.69 mg/L), significantly higher (*p* < 0.05) than the other strains. The dissolved potassium content ranged from 2.52 mg/L to 6.20 mg/L. Strains DX3 and DX37 showed significantly superior (*p* < 0.05) potassium dissolution (6.14 mg/L and 6.20 mg/L, respectively) compared to the rest. Analysis of broth pH during these assays (Figure 11D) revealed distinct patterns: During organic phosphate solubilization, the broth pH for strains X1 and Y8 was significantly higher (*p* < 0.05) than the control (CK), whereas the pH for the other six strains was significantly lower (*p* < 0.05) than CK. During inorganic phosphate solubilization and potassium dissolution, the broth pH for all eight strains was significantly lower (*p* < 0.05) than the CK.

**Figure 12:**
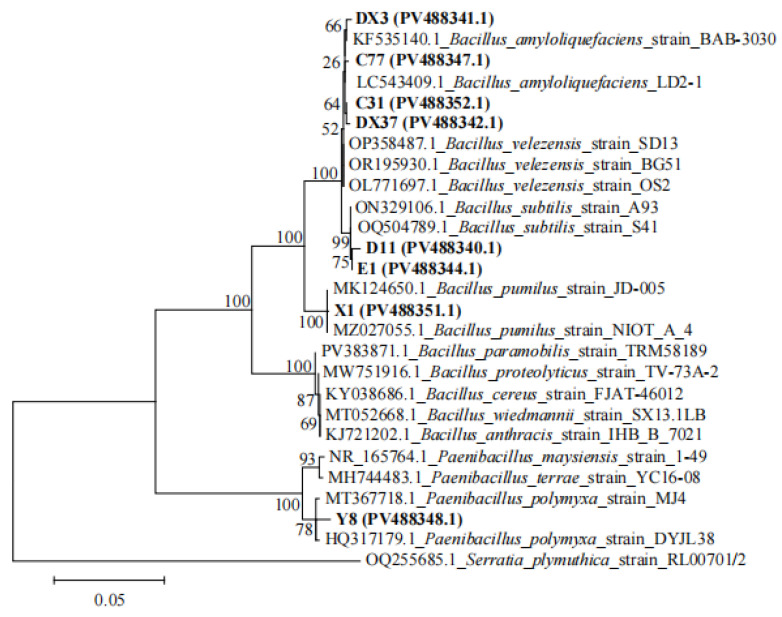
Maximum likelihood phylogenetic tree of beneficial bacterial strains based on 16S rRNA gene sequences. Bootstrap values are indicated at branch nodes. GenBank accession numbers are shown in parentheses. Scale bar = 0.05 nucleotide substitutions per site.

Phylogenetic analysis based on 16S rRNA gene sequences was performed to taxonomically classify the isolated PGPR strains (Figure 12). Isolates DX3 and C77 clustered within a clade containing reference strain KF535140.1 and LC543409.1 (all *B. amyloliquefaciens*). Combined with morphological characteristics, these isolates were preliminary identification as *B*. *amyloliquefaciens*. Isolates C31 and DX37 exhibited close phylogenetic affiliation with reference strains OP358487.1, OR195930.1, and OL771697.1 (all *B. velezensis*). Combined with morphological characteristics, these isolates were preliminary identification as *B*. *velezensis*. Isolates D11 and E1 exhibited close phylogenetic affiliation with reference strains ON329106.1 and OQ504789.1 (all *B. subtilis*). Combined with morphological characteristics, these isolates were preliminary identification as *Bacillus subtilis*. Isolate X1 exhibited close phylogenetic affiliation with reference strains MK124650.1 and MZ027055.1 (all *B. pumilus*), supporting its preliminary identification as *Bacillus pumilus*. Isolate Y8 clustered closely with reference strains MT367718.1 and HQ317179.1 (all *P. polymyxa*). Morphological congruence underpinned its preliminary identification as *Paenibacillus polymyxa*. The GenBank accession numbers of all isolated strains are labeled on the phylogenetic tree.

### 3.8. Antagonistic Activity of Rhizosphere Beneficial Microorganisms Against Pathogens

**Figure 13:**
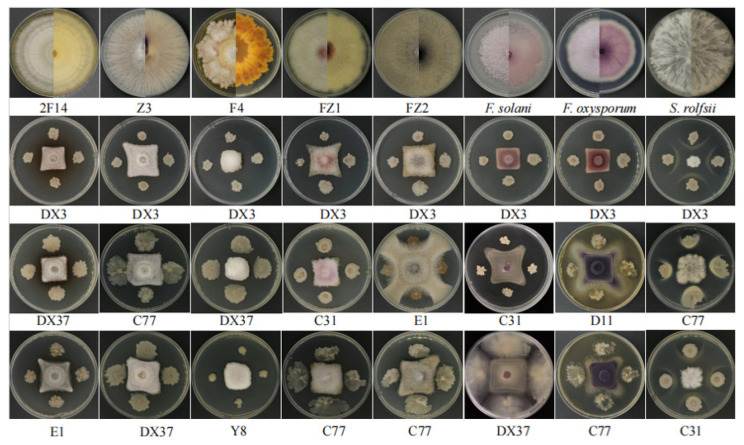
Antagonistic activity of seven beneficial bacterial strains against *A. carmichaelii*-associated pathogens and reference phytopathogens. Panel rows: Row 1—Control growth of pathogens without bacterial antagonists. Rows 2–4—Pathogen growth inhibition by beneficial bacteria. All cultures incubated at 28 °C for 7 days.

Antagonistic assays against phytopathogens revealed that seven of the eight PGPR strains exhibited antifungal activity (Figure 13), while all eight demonstrated antibacterial activity against *S. marcescens* L23 (Figure 9). Strain DX3 (*B. amyloliquefaciens*) exhibited the strongest inhibition (> 65%, *p* < 0.05) against isolated potential pathogens (2F14 [*F. solani*], Z3 [*C. rosea*], F4 [*F. avenaceum*], FZ1 [*M. hiemalis*], and FZ2 [*M. racemosus*]) and reference phytopathogens (*F. solani*, *F. oxysporum*, and *S. rolfsii*) (Figure 13, Table 2). This broad-spectrum antagonism highlights DX3’s potent biocontrol capability. Consequently, DX3 was selected for subsequent disease control trials based on its exceptional efficacy.

### 3.9. Quantification of Growth-Promoting Effects by Rhizosphere Beneficial Microorganisms

**Figure 14:**
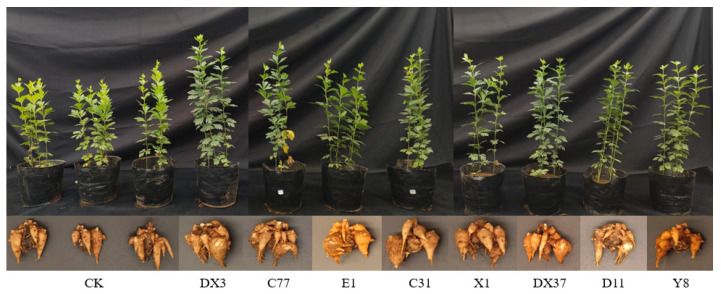
Growth promotion of *A. carmichaelii* caused by eight plant growth-promoting *Bacillus* strains.

Pot trial results demonstrated that at 60 days post-inoculation, the treatment groups exhibited significantly greater plant height, stem diameter, SPAD value (indicative of chlorophyll content), fresh shoot weight, and fresh tuber weight compared to the control group (Figure 14, Table 3). All eight inoculated strains promoted the growth of *A. carmichaelii*, with strain DX3 inducing the most pronounced overall growth promotion. Relative to the control, DX3 inoculation resulted in significant increases (*p* < 0.05) of 26.73%, 36.43%, 36.93%, 59.89%, 94.95%, and 145.42% in plant height, stem diameter, leaf number, chlorophyll content, fresh shoot weight, and fresh tuber weight, respectively. Furthermore, we analyzed the physicochemical properties of rhizosphere soil following inoculation with each strain. As presented in Table 4, all eight strains reduced soil pH, enhanced the availability of key nutrients (AN, AP, AK), and increased OM content. Specifically, pH decrease ranged from 0.02 to 0.22 units, while AN, AP, AK, and OM increased by 2.39–19.44%, 3.37–39.25%, 10.54–57.93%, and 3.70–14.47%, respectively. Notably, strain X1 exhibited the most significant improvements: compared to the control, it reduced pH by 0.22 units (*p* < 0.05), while increasing AN (19.44%), AP (36.91%), AK (57.93%), and OM (14.47%) (*p* < 0.05).

### 3.10. Biocontrol Efficacy of Strain DX3 Against A. carmichaelii Root Rot

**Figure 15:**
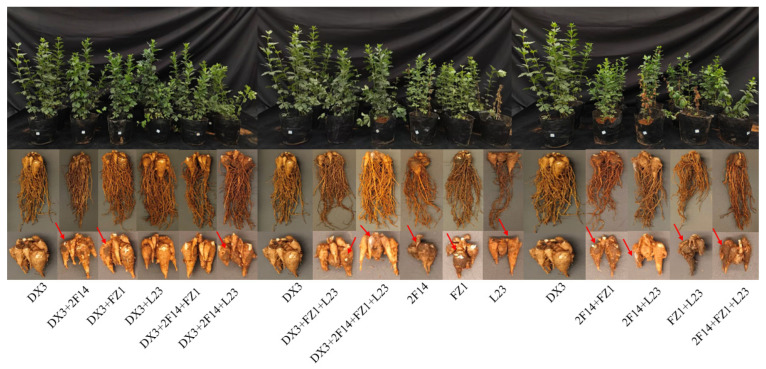
Biocontrol efficacy of strain DX3 against *A. carmichaelii* root rot in pot trials. Red arrows indicate characteristic disease symptoms on root tubers.

The efficacy of biocontrol strain DX3 against the potential pathogens 2F14 (*F. solani*), FZ1 (*M. hiemalis*), and L23 (*S. marcescens*) in pot trials is shown in Figure 15. Compared to plants inoculated solely with pathogens, those pre-treated with DX3 exhibited no overt disease symptoms, with normal tuber expansion and only mild signs of infection. Conversely, plants inoculated only with pathogens displayed severe root rot symptoms, including stunted growth, leaf yellowing, and even complete wilting in some individuals. Pathogen-infected tubers were smaller, severely affected by disease, and, in some cases, extensively decayed with white mycelial coverage on the surface. Analysis of disease severity and biocontrol efficacy across treatments (Table 5 and Table 6) revealed the following: The disease severity index (DSI) ranged significantly (*p* < 0.05) from 39.55% to 60.44%. Inoculation with L23 (*S. marcescens*) alone or the combination FZ1 + L23 (*M. hiemalis* + *S. marcescens*) induced significantly higher (*p* < 0.05) DSI than other single or combined pathogen treatments. Notably, no direct correlation was observed between the pathogen inoculation method (single vs. combined) and disease incidence. Pre-inoculation with DX3 significantly reduced (*p* < 0.05) the DSI to a range of 18.22–21.33%. The biocontrol efficacy of DX3 consistently exceeded 50% against all pathogen treatments. Significantly, its efficacy against the FZ1 + L23 combination reached 69.10%, markedly higher (*p* < 0.05) than against other treatments. Furthermore, DX3 inoculation significantly enhanced (*p* < 0.05) the activity of key plant defense enzymes (Table 6). The most pronounced induction occurred in plants challenged with L23 (*S. marcescens*), where DX3 treatment led to remarkable increases of 180.22%, 35.97%, and 254.91% in polyphenol oxidase (PPO), peroxidase (POD), and phenylalanine ammonia-lyase (PAL) activity, respectively, compared to pathogen-only controls. These results collectively demonstrate that DX3 effectively boosts the activity of these defense enzymes in *A. carmichaelii*, thereby enhancing plant resistance to root rot.

## 4. Discussion

The primary constraint in *A. carmichaelii* production is the exacerbation of soil-borne diseases, with root rot representing one of the most severe pathologies [40]. Multiple pathogenic microorganisms have been implicated in *A. carmichaelii* root diseases. Identifying the causative agents is therefore critical for safeguarding plant health. *Bacillus* spp. have garnered significant research interest in recent years owing to their dual plant growth-promoting and biocontrol properties, demonstrating considerable potential for agricultural applications [41,42]. In this study, it was isolated 15 putative pathogens from the rhizosphere and root tubers of diseased plants that significantly contributed to tuber rot. These strains encompassed eight species across four genera. Concurrently, eight PGP strains were isolated from the rhizosphere of healthy plants, all taxonomically identified as *Bacillus* spp. Notably, seven of these strains exhibited pronounced antagonistic activity against both the isolated potential pathogens and known pathogenic agents associated with *A. carmichaelii* root rot. Healthy soil ecosystems play a vital role in enhancing crop yield, bolstering resistance to biotic and abiotic stresses, and maintaining functional rhizosphere microbial communities [43]. Conversely, continuous monoculture can disrupt rhizosphere microbiota equilibrium, impede nutrient cycling, and exacerbate soil acidification [44]. Consequently, comprehensive analysis of soil physicochemical properties and the microbial community composition in both *A. carmichaelii* and its rhizosphere is imperative for developing effective biocontrol strategies against root rot. This investigation employed a multi-compartment comparative approach, analyzing differences in rhizosphere soil properties and microbial communities (encompassing rhizosphere soil, root tuber interior, epidermis, and fibrous roots) between healthy and diseased plants. This integrated methodology provides critical insights for elucidating key etiological factors of *A. carmichaelii* root rot.

Microbial diversity positively contributes to functional diversity within soil ecosystems [45]. Comparative analyses between healthy and diseased soils reveal that shifts in microbial community composition often coincide with alterations in keystone species, suggesting concomitant functional modifications [46]. This underscores the intrinsic linkage between community dynamics and ecosystem processes. Long-term monoculture can induce fundamental changes in soil physicochemical properties, potentially driving broad functional transitions [47]. In this study, significantly higher Chao1 and Shannon indices were observed in the rhizosphere, epidermis, and root tuber interior of healthy *A. carmichaelii* compared to diseased plants. Notably, rhizospheric microbial diversity substantially exceeded that of other compartments across both health statuses (*p* < 0.05). Distinct microbial community structures were evident among plant compartments and between health conditions, indicating that diversity loss and structural disruption constitute key etiological factors in root rot pathogenesis. Community composition analysis revealed dramatic reductions in the relative abundance of *Bacillus* and *Mortierella* in diseased plants. Healthy root tuber epidermis uniquely enriched *Beauveria*, whereas diseased tuber interiors were dominated by *Erwinia* (59.34% relative abundance). These findings suggest that dysbiosis in beneficial taxa (*Bacillus*, *Mortierella*, and *Beauveria*) may predispose plants to disease. This aligns with studies on *Lycium barbarum* root rot, where healthy/diseased plants exhibited divergent dominant microbiota across rhizoplane, rhizosphere, and root zone compartments, with functional predictions implicating *Fusarium* as a key pathogen [48]. Plant health is intrinsically linked to soil physicochemical and microbiological properties [49]. It was identified significant differences between healthy and diseased rhizospheres, namely that diseased soils showed reduced pH and AN, but elevated TN, TP, TK, AP, AK, and OM (*p* < 0.05). Correlation analysis demonstrated strong associations between healthy plant microbiomes and critical soil properties (pH, AN, and OM). Notably, *Bacillus*, *Mortierella*, and *Beauveria* diminished in diseased plants are well-documented beneficial genera [50,51,52]. These taxa enhance soil fertility through bioactive metabolite production (e.g., antibiotics, phytohormones, extracellular enzymes), directly contributing to plant growth promotion and systemic resistance induction. Their depletion likely compromises soil functionality, creating conditions conducive to pathogen dominance. Based on the integrated analysis of microbial community data, it was further inferred that the relative abundances of *Bacillus*, *Mortierella*, and *Beauveria* were lower in diseased *A. carmichaelii* plants compared to healthy ones, and in some cases, these microbial taxa were even absent. The downregulation or loss of these beneficial microorganisms may lead to severe dysregulation of soil physicochemical properties, thereby contributing to disease onset.

Plant–microbe interactions exhibit inherent complexity, necessitating rigorous pathogen identification to elucidate key etiological agents of *A. carmichaelii* root rot. The isolation of putative pathogens from diseased rhizospheres and tissues, followed by validation through Koch’s postulates [53], provides critical mechanistic insights. In this study, it is inferred that *F. solani*, *F. avenaceum*, *C. rosea*, *M. racemosus*, *M. irregularis*, *M. hiemalis*, *S. liquefaciens*, and *S. marcescens* may be potential pathogenic agents causing root rot in *A. carmichaelii*. Notably, the extant literature documents *A. carmichaelii* root diseases caused by diverse pathogens including *F. solani*, *F. oxysporum*, *S. rolfsii*, *I. robusta*, *P. brasiliense*, *P. aeruginosa*, and *S. marcescens* [16,17,18,19]. This polyetiological paradigm indicates that *A. carmichaelii* root rot arises not from monoinfection but through complex infections involving multiple pathogens. Studies have indicated that *Mucor* can cause decay in *Codonopsis pilosula* [54], and the occurrence of symptoms is associated with multiple pathogens. Root rot of *Angelica sinensis* and *Astragalus membranaceus* caused by *C. rosea* has been reported in China [55,56], and root rot of *Gastrodia elata* caused by *C. rosea* has also been reported in South Korea [57]. Based on previous research findings, *F. solani* and *S. marcescens* isolated in this study were confirmed to be among the key factors causing diseases in *A. carmichaelii*, while *F. avenaceum*, *C. rosea*, *S. liquefaciens*, and three *Mucor* species (*M. racemosus*, *M. irregularis*, and *M. hiemalis*) may also be potential pathogenic microorganisms contributing to *A. carmichaelii* diseases. However, the results of subsequent isolation and validation of these potential pathogens were not entirely consistent with the microbial diversity analysis, which may be attributed to limitations in the detection methods and randomness in the isolation process. This discrepancy warrants further investigation using more advanced methodologies.

Building upon the identification of etiological agents, it was isolated eight *Bacillus* strains from the healthy *A. carmichaelii* rhizosphere exhibiting dual plant growth-promoting and biocontrol functions. These strains universally demonstrated capabilities for IAA and siderophore production, phosphate solubilization (organic/inorganic), potassium dissolution, nitrogen fixation, and cellulose degradation. Crucially, they displayed antagonistic activity against both reference pathogens (*F. solani*, *F. oxysporum*, and *S. rolfsii*) and our isolated potential pathogens. The functional efficacy of beneficial microbes hinges on their ability to mobilize recalcitrant nutrients like phosphates [58]. Quantitative assays revealed strain-specific variations in IAA production (13.11–26.79 mg/L), organic phosphate solubilization (15.10–63.98 mg/L), inorganic phosphate solubilization (69.80–270.69 mg/L), and potassium dissolution (2.52–6.20 mg/L). Notably, pH dynamics diverged during solubilization. Concerning the acid-mediated mechanism, most strains decreased broth pH during solubilization, indicative of organic acid secretion [59], while for the alkaline phosphatase pathway, strains X1 and Y8 increased pH during organic phosphate solubilization, suggesting enzymatic hydrolysis via alkaline phosphatases [60]. This functional heterogeneity underscores strain-specific mechanisms. PGPR enhances yield by improving soil fertility and restructuring microbial communities [61]. Our pot trials confirmed that all eight *Bacillus* strains significantly improved agronomic traits and biomass in *A. carmichaelii*, likely through mobilizing bioavailable N/P/K nutrients [62]. Preemptive inoculation with DX3 (*B. amyloliquefaciens*) significantly reduced the disease index and potently induced defense enzymes (PPO, POD, and PAL), demonstrating effective biocontrol. This aligns with studies where *B. amyloliquefaciens*, *B. amyloliquefaciens* Oj-2.16, and *B. velezensis* controlled anthracnose in loquat by activating defense enzymes [63], enhanced CAT/SOD/POD/PAL to suppress *Verticillium wilt* in tomato [64], and primed defense responses against *Fusarium* in maize [65], respectively. Our findings establish that *B. amyloliquefaciens* DX3 confers protection via growth promotion and systemic resistance induction in *A. carmichaelii*.

Identifying key pathogens and developing green control strategies are pivotal for sustainable production. In this experiment, the wound inoculation method was primarily used on sliced *A. carmichaelii* tissues to verify the pathogenicity of the isolated microbes. This approach may have certain limitations. Some potentially pathogenic isolates were selected for pot-based pathogenicity assays, and the results were acceptable. However, further field validation is required for these isolates to better elucidate their pathogenic mechanisms. Additionally, the *Bacillus* strains obtained in this study exhibit growth-promoting and biocontrol potential, and they can be further tested and developed in combination with field trials to obtain effective microbial inoculants for improving the yield and quality of *A. carmichaelii*.

## 5. Conclusions

*A. carmichaelii* root rot induces significant alterations in bacterial and fungal community diversity/composition across rhizosphere soil, root tuber epidermis, tuber interior, and fibrous roots, concomitant with modified rhizosphere physicochemical properties. Pathogenesis correlates strongly with dysbiosis of beneficial taxa (*Bacillus*, *Mortierella*, *Beauveria*, and *Tausonia*). It was identified eight putative pathogens: *F. solani*, *F. avenaceum*, *C. rosea*, *M. racemosus*, *M. irregularis*, *M. hiemalis*, *S. liquefaciens*, and *S. marcescens*. To develop green control strategies, isolated *Bacillus* strains from healthy rhizospheres exhibiting multifunctional traits, namely synthesis of IAA and siderophores, nitrogen fixation and cellulose degradation, solubilization of organic/inorganic phosphates, potassium dissolution, and broad-spectrum antagonism against pathogens, were involved. These strains significantly enhanced plant growth and tuber expansion (>145% biomass increase) while suppressing disease incidence (>50% efficacy). Mechanistic studies revealed that their biocontrol efficacy operates through restoring beneficial microbial consortia, activating soil N/P/K pools, and upregulating defense enzymes (PPO/POD/PAL). This work provides both theoretical foundations for rhizosphere microbiome management and practical microbial resources for developing sustainable *A. carmichaelii* protection strategies.

## Figures and Tables

**Table 1:** Rhizosphere soil properties of healthy and diseased *A. carmichaelii* plants.

Treatment	pH	TN (g/Kg)	TP (g/Kg)	TK (g/Kg)	AN (mg/Kg)	AP (mg/Kg)	AK (mg/Kg)	OM (g/Kg)
HT	6.66 ± 0.06 a	4.68 ± 0.04 b	2.05 ± 0.15 b	1.96 ± 0.06 b	549.20 ± 5.40 a	4.48 ± 0.31 b	395.20 ± 20.33 b	99.20 ± 3.56 b
IT	5.52 ± 0.19 b	5.03 ± 0.07 a	2.36 ± 0.13 a	2.24 ± 0.05 a	505.60 ± 3.20 b	7.44 ± 0.29 a	1611.00 ± 61.89 a	118.40 ± 3.85 a

Note: Significant differences are marked with letters (*p* < 0.05).

**Table 2:** Inhibition rates (%) of seven beneficial bacterial strains against *A. carmichaelii*-associated potential pathogens and reference phytopathogens (*F. solani*, *F. oxysporum*, and *S. rolfsii*).

Pathogen Inhibition Rate (%)								
Strain	2F14	FZ1	Z3	F4	FZ2	*Fusarium solani*	*Fusarium oxysporum*	*Sclerotium rolfsii*
C77	67.27 ± 1.50 cd	66.25 ± 2.43 a	61.58 ± 1.00 b	64.31 ± 1.89 bc	63.27 ± 2.12 ab	56.58 ± 1.19 cd	59.91 ± 1.04 b	66.50 ± 2.19 c
C31	71.17 ± 1.55 ab	67.54 ± 1.97 a	61.91 ± 1.20 ab	61.46 ± 3.33 cd	60.67 ± 2.19 bc	58.02 ± 0.80 c	62.78 ± 2.50 b	72.33 ± 1.66 b
D11	61.81 ± 0.70 f	51.77 ± 1.75 b	53.96 ± 0.75 c	52.92 ± 2.86 e	55.77 ± 1.86 d	55.08 ± 1.02 cd	43.07 ± 3.00 e	/
DX3	72.80 ± 1.28 a	68.23 ± 0.66 a	65.10 ± 3.98 a	70.64 ± 0.50 a	65.79 ± 1.33 a	72.75 ± 1.26 a	67.82 ± 2.48 a	83.09 ± 2.91 a
DX37	69.28 ± 1.60 bc	65.66 ± 1.86 a	62.10 ± 0.69 ab	67.41 ± 2.63 ab	65.41 ± 0.72 a	65.11 ± 0.64 b	60.30 ± 1.99 b	50.44 ± 1.86 d
E1	65.17 ± 1.80 de	55.29 ± 1.46 b	56.41 ± 1.52 c	59.95 ± 1.36 cd	57.24 ± 2.73 cd	52.93 ± 2.69 d	48.15 ± 1.05 d	52.76 ± 0.95 d
Y8	63.69 ± 2.27 ef	54.17 ± 2.92 b	54.02 ± 0.57 c	58.71 ± 3.56 d	46.22 ± 3.29 e	54.03 ± 5.81 cd	55.68 ± 2.97 c	/

Note: Significant differences are marked with letters (*p* < 0.05).

**Table 3:** Growth-promoting effects of eight beneficial bacterial strains on agronomic traits and biomass of *A. carmichaelii.*

Treatment	Plant Height (cm)	Stem Diameter (cm)	Leaf Number (count)	Chlorophyll Content (SPAD)	Fresh Shoot Weight (g)	Fresh Tuber Weight (g)
CK	54.69 ± 3.69 b	2.80 ± 0.24 d	11.67 ± 1.53 d	33.33 ± 1.17 b	14.66 ± 1.96 c	15.08 ± 4.02 c
DX3	69.31 ± 4.05 a	3.82 ± 0.29 bc	16.33 ± 1.53 a	53.29 ± 4.62 a	28.58 ± 4.55 a	37.01 ± 9.60 a
C77	67.67 ± 6.60 a	3.64 ± 0.33 bc	14.00 ± 1.00 abcd	49.91 ± 4.90 a	20.34 ± 2.86 b	26.43 ± 3.45 b
E1	63.35 ± 1.60 a	3.52 ± 0.13 c	13.33 ± 0.58 cd	45.01 ± 4.13 a	20.77 ± 2.83 b	26.65 ± 2.14 b
C31	68.61 ± 5.02 a	4.08 ± 0.34 b	15.00 ± 1.00 abc	48.51 ± 5.89 a	21.24 ± 2.94 b	26.33 ± 3.40 b
X1	67.20 ± 3.50 a	3.38 ± 0.18 c	13.67 ± 0.58 bcd	50.18 ± 1.53 a	20.59 ± 3.49 b	23.75 ± 4.87 b
DX37	66.58 ± 4.12 a	3.63 ± 0.31 bc	14.33 ± 1.15 abc	51.59 ± 8.96 a	23.78 ± 2.49 ab	26.33 ± 2.99 b
D11	66.37 ± 5.86 a	4.73 ± 0.38 a	16.00 ± 2.65 ab	53.24 ± 4.05 a	20.75 ± 4.63 b	26.15 ± 5.74 b
Y8	70.40 ± 1.53 a	3.85 ± 0.18 bc	14.33 ± 0.58 abc	50.66 ± 1.16 a	21.03 ± 1.55 b	21.04 ± 1.84 bc

Note: Significant differences are marked with letters (*p* < 0.05).

**Table 4:** Effects of eight beneficial bacterial strains on the physicochemical properties of the rhizosphere soil of *A. carmichaelii.*

Treatment	pH	AN (mg/Kg)	AP (mg/Kg)	AK (mg/Kg)	OM (g/Kg)
CK	7.36 ± 0.04 a	108.22 ± 5.36 c	16.61 ± 1.40 b	221.69 ± 5.78 f	35.17 ± 0.57 c
DX3	7.19 ± 0.03 ef	125.97 ± 2.13 ab	20.82 ± 1.66 a	335.74 ± 14.38 a	39.16 ± 1.64 ab
C77	7.23 ± 0.02 de	120.00 ± 1.58 b	22.73 ± 1.23 a	309.66 ± 17.52 bc	40.13 ± 1.55 a
E1	7.29 ± 0.02 bc	124.77 ± 3.78 ab	23.13 ± 1.60 a	330.77 ± 12.73 ab	36.47 ± 1.49 bc
C31	7.25 ± 0.03 cd	122.51 ± 4.72 ab	18.07 ± 0.62 b	259.51 ± 9.46 e	37.87 ± 2.65 abc
X1	7.14 ± 0.03 f	129.26 ± 2.66 a	22.74 ± 0.86 a	350.12 ± 14.91 a	40.26 ± 1.12 a
DX37	7.24 ± 0.04 de	110.81 ± 3.60 c	23.01 ± 1.29 a	293.98 ± 9.55 cd	38.18 ± 0.95 ab
D11	7.26 ± 0.03 cd	113.45 ± 2.89 c	17.72 ± 1.32 b	284.34 ± 14.07 d	39.04 ± 1.63 ab
Y8	7.34 ± 0.04 ab	129.17 ± 5.17 a	17.17 ± 0.73 b	245.05 ± 10.18 e	38.68 ± 1.25 ab

Note: Significant differences are marked with letters (*p* < 0.05).

**Table 5:** Suppression of *A. carmichaelii* root rot by strain DX3 in pot trials.

Treatment	Disease Severity Index (DSI) (%)	Treatment	Disease Severity Index (DSI) (%)	Biocontrol Efficacy (BE) (%)
2F14	53.78 ± 2.04 b	DX3+2F14	19.78 ± 3.01 a	63.32 ± 4.27 ab
FZ1	46.22 ± 3.08 c	DX3+FZ1	20.00 ± 4.00 a	56.60 ± 8.86 b
L23	60.44 ± 2.78 a	DX3+L23	21.33 ± 3.53 a	64.63 ± 6.19 ab
2F14+FZ1	40.00 ± 1.33 d	DX3+2F14+FZ1	18.22 ± 2.04 a	54.48 ± 4.32 b
2F14+L23	46.67 ± 4.81 c	DX3+2F14+L23	18.44 ± 2.52 a	60.41 ± 4.21 ab
FZ1+L23	59.55 ± 2.04 a	DX3+FZ1+L23	18.45 ± 3.67 a	69.10 ± 5.39 a
2F14+FZ1+L23	39.55 ± 2.04 d	DX3+2F14+FZ1+L23	18.67 ± 3.53 a	52.84 ± 8.26 b

Note: Significant differences are marked with letters (*p* < 0.05).

**Table 6:** Effects of strain DX3 inoculation on key defense enzyme activities (POD, PPO, and PAL) in *A. carmichaelii* plants challenged with pathogens.

Treatment	PPO (U/g)	POD (U/g)	PAL (U/g)
2F14	208.87 ± 29.00 ef	1672.70 ± 132.02 c	35.04 ± 5.53 de
FZ1	259.52 ± 27.94 de	1701.38 ± 45.44 c	42.67 ± 4.76 d
L23	187.27 ± 28.16 f	1580.15 ± 97.37 c	27.50 ± 6.03 e
2F14+FZ1	285.62 ± 30.79 d	1545.22 ± 59.32 c	34.76 ± 5.16 de
2F14+L23	295.46 ± 30.39 d	1707.44 ± 49.53 c	32.53 ± 5.28 de
FZ1+L23	218.81 ± 40.50 ef	1522.61 ± 132.43 c	26.98 ± 4.60 e
2F14+FZ1+L23	221.36 ± 18.41 ef	1666.23 ± 134.14 c	43.423.40 d
DX3+2F14	539.76 ± 37.45 b	2160.38 ± 66.68 ab	85.02 ± 10.01 b
DX3+FZ1	593.65 ± 27.79 a	2243.80 ± 52.92 a	71.10 ± 4.66 c
DX3+L23	524.77 ± 31.23 b	2148.51 ± 89.83 ab	97.60 ± 5.72 a
DX3+2F14+FZ1	555.40 ± 41.65 ab	2018.11 ± 88.50 b	76.33 ± 7.32 bc
DX3+2F14+L23	507.31 ± 11.66 bc	2042.32 ± 153.50 ab	76.92 ± 7.03 bc
DX3+FZ1+L23	458.57 ± 30.89 c	2182.10 ± 127.96 ab	97.86 ± 6.44 a
DX3+2F14+FZ1+L23	467.26 ± 22.76 c	2187.23 ± 204.51 ab	72.17 ± 7.84 c

Note: Significant differences are marked with letters (*p* < 0.05).

## Data Availability

The data presented in this study are available on request from the corresponding authors.
